# Singlet Molecular Oxygen Generation by Light-Activated DHN-Melanin of the Fungal Pathogen *Mycosphaerella fijiensis* in Black Sigatoka Disease of Bananas

**DOI:** 10.1371/journal.pone.0091616

**Published:** 2014-03-19

**Authors:** Miguel J. Beltrán-García, Fernanda M. Prado, Marilene S. Oliveira, David Ortiz-Mendoza, Alexsandra C. Scalfo, Adalberto Pessoa, Marisa H. G. Medeiros, James F. White, Paolo Di Mascio

**Affiliations:** 1 Departamento de Química-ICET, Universidad Autónoma de Guadalajara, Zapopan Jalisco, Mexico; 2 Departamento de Bioquímica, Instituto de Química, Universidade de São Paulo, São Paulo, SP, Brazil; 3 Instituto de Ingeniería, Universidad Autónoma de Baja California, Mexicali Baja California, Mexico; 4 Faculdade de Ciências Farmacêuticas, Departamento de Tecnologia Bioquímico-Farmacêutica, Universidade de São Paulo, São Paulo, Brazil; 5 Department of Plant Biology and Pathology, School of Environmental and Biological Sciences, Rutgers University, New Brunswick, New Jersey, United States of America; Juntendo University School of Medicine, Japan

## Abstract

In pathogenic fungi, melanin contributes to virulence, allowing tissue invasion and inactivation of the plant defence system, but has never been implicated as a factor for host cell death, or as a light-activated phytotoxin. Our research shows that melanin synthesized by the fungal banana pathogen *Mycosphaerella fijiensis* acts as a virulence factor through the photogeneration of singlet molecular oxygen O_2_ (^1^Δ_g_). Using analytical tools, including elemental analysis, ultraviolet/infrared absorption spectrophometry and MALDI-TOF mass spectrometry analysis, we characterized both pigment content in mycelia and secreted to the culture media as 1,8-dihydroxynaphthalene (DHN)-melanin type compound. This is sole melanin-type in *M. fijiensis*. Isolated melanins irradiated with a Nd:YAG laser at 532 nm produced monomol light emission at 1270 nm, confirming generation of O_2_ (^1^Δ_g_), a highly reactive oxygen specie (ROS) that causes cellular death by reacting with all cellular macromolecules. Intermediary polyketides accumulated in culture media by using tricyclazole and pyroquilon (two inhibitors of DHN-melanin synthesis) were identified by ESI-HPLC-MS/MS. Additionally, irradiation at 532 nm of that mixture of compounds and whole melanized mycelium also generated O_2_ (^1^Δ_g_). A pigmented-strain generated more O_2_ (^1^Δ_g_) than a strain with low melanin content. Banana leaves of cultivar Cavendish, naturally infected with different stages of black Sigatoka disease, were collected from field. Direct staining of the naturally infected leaf tissues showed the presence of melanin that was positively correlated to the disease stage. We also found hydrogen peroxide (H_2_O_2_) but we cannot distinguish the source. Our results suggest that O_2_ (^1^Δ_g_) photogenerated by DHN-melanin may be involved in the destructive effects of *Mycosphaerella fijiensis* on banana leaf tissues. Further studies are needed to fully evaluate contributions of melanin-mediated ROS to microbial pathogenesis.

## Introduction

The term “melanin” encompasses a heterogeneous group of polymeric amorphous substances without a defined structure, that share properties of being black or brown to red in colour, highly insoluble in water and organic solvents, susceptible to bleaching by oxidizing agents like hydrogen peroxide (H_2_O_2_), hypochlorite ion (OCl^-^) and having a featureless absorption spectrum from the far UV (ultraviolet) to the infrared (IR) region. Melanin is a unique pigment with many functions in animals, plants, bacteria and fungi. Three types of melanins occur naturally: eumelanins and pheomelanins derived from DOPA (dihydroxyphenylalanine) and allomelanins formed through oxidation and polymerization of 1,8-dihydroxynaphthalene (DHN). Eumelanins contain nitrogen atoms, pheomelanin contains nitrogen and sulphur atoms and allomelanins contain neither. The association of melanin production with protection against UV light is generally accepted [1], [2]. For microorganisms, melanin participates in energy transduction and electron transfer processes [3], [4]. On the other hand eumelanin was found to act as a photosensitizers under UV radiation, thereby generating reactive oxygen species (ROS) such as hydrogen peroxide (H_2_O_2_), hydroxyl radical (OH) [5] and singlet molecular oxygen [O_2_ (^1^Δ_g_)] [6], with some studies implicating melanin photochemistry with the production of DNA strand breaks [7]. However melanin also acts as a scavenger of a variety of oxidizing and reducing radicals [8].

In the fungal kingdom, the ascomycetous fungi generally produce 1,8-DHN-melanin-type, although *Aspergillus* produces DOPA-melanin [9]. For basidiomycetous fungi, the pigment is derived from phenolic precursors as glutaminyl-3,4-dihydroxybenzene (GDBH) or catechol. The pathogenic yeast *Cryptococcus neoformans* produces DOPA-melanin when dihydroxyphenylalanine compounds are present in the culture medium in which tyrosinases and laccases hydroxylate tyrosine to DOPA to dopaquinone [10]. Melanins are typically localized in cell walls where they are likely cross-linked to polysaccharides (mainly chitin), and sometimes excreted into the medium as soluble extracellular polymers. The 1,8-DHN-melanin pigment is synthesized from acetyl-coA or malonyl-CoA, and formation of 1,3,6,8-tetrahydroxynaphthalene (1,3,6,8-THN) is catalysed by a polyketide synthase (PKS). After reduction and dehydration reactions the intermediates scytalone, 1,3,8-trihydroxynaphthalene (1,3,8-THN), vermelone and finally 1,8-DHN are produced; and melanin forms by an oxidative polymerization of 1,8-DHN catalysed by phenoloxidases [11]. These pigments are not considered essential for fungal growth and development, but enhance fungal survival and competitive abilities in extreme environments better than related non-pigmented fungal strains. *In vitro* studies have shown that melanised fungi resist extreme temperatures, desiccation, ionizing radiation, plant defence mechanisms, hydrolytic enzymes, ROS, and heavy metal toxicity. Melanin itself is a powerful cation chelator [12], [13]. For fungal pathogens, melanin contributes to virulence in humans as well as plants. Melanin provides protection from host defence mechanisms involving oxidizing agents and protects sclerotia, conidia or other melanized structures from lysis [12]. In the plant pathogens *Magnaphorthe grisea*, *Colletrotrichum* species, *Venturia inaequalis*, and *Diplocarpon rosae* melanin is critical to host invasion. These fungi produce appresoria, that require melanin to sustain turgor pressure to penetrate host leaves [14], [15], [16]. Melanin also impacts the overall porosity of the cell wall. The reduction in pore size combined with the absorption properties of melanin are suggested as a mechanism for acquired fungicide resistance [17].

The hemibiotrophic fungus *Mycosphaerella fijiensis* Morelet (sexual phase) or *Pseudocercospora fijiensis* (Morelet) Deighton (asexual phase) is a plant pathogen of banana and plantains, causing black leaf streak also called black Sigatoka. This fungus is responsible for more than 50% of the crop losses in productions areas. *M. fijiensis* shows high levels of genetic diversity, aggressiveness and resistance to fungicides and ROS [18], [19], [20]. This fungus accumulates and secretes a dark-green pigment on the surface of the colony and into potato-dextrose agar. When the fungus is grown in a liquid medium, it forms dark mycelial pellets and the medium becomes dark after 6 days of incubation. This increased dark coloration is related age of the culture. Isogenic mutants of *M. fijiensis* that display a pink pigmentation in mycelium and very low melanin content are able to penetrate banana leaf tissue, but infection is blocked at early stages and necrotic lesions that form on leaves are suggested to result from hypersensitive defence response of the host [21]. As a result of these observations, we hypothesized that melanin itself was involved in the process that induced extensive necrosis and cell death in plant tissues infected by the black Sigatoka pathogen.

There are a growing number of publications on 1,8-DHN fungal melanins, but these have been focused mainly on the characterization of the genes involved in the synthesis pathway, spectrophotometric characterization, ultra-structural localization, measuring antioxidant capacity and the pathogenic behaviour of melanin deficient strains on their hosts. To this last point it has been proposed that fungal melanin acts as an antioxidant agent against host defence mechanisms [22], [23]. Herein we studied the melanin pigment of *M. fijiensis* accumulated at cell walls and secreted into the culture medium, through applications of spectrophotometric techniques like UV, IR and elemental analysis and Matrix-Assisted Laser Desorption/Ionization-Time of Flight (MALDI-TOF) mass spectrometry. We identified both secreted and non-secreted pigments as 1,8-DHN-melanin. Moreover, generation ^1^O_2_ (Δg) was investigated for *M. fijiensis* melanins, and for intermediate products generated in the presence of melanin biosynthesis inhibitors (tricyclazole and pyroquilon) by flash photolysis with Nd:YAG laser at 532 nm. The identification by High-Performance Liquid Chromatography coupled to mass spectrometry in tandem with electrospray ionization source (ESI-HPLC-MS/MS) of intermediate products in the liquid cultures blocked by tricyclazole and pyroquilon confirming the generation of O_2_ (^1^Δ_g_). The presence of melanin in naturally infected leaf tissue was positively related to the disease stage. Our results suggest that the melanin produced by *M. fijiensis* generates O_2_ (^1^Δ_g_) that may function as a “photoactivated toxin” that triggers cell death in infected leaves, resulting in the destructive symptoms of black Sigatoka disease in bananas and plantains.

## Results

### The Green Black Pigment of *M. fijiensis* is a 1,8-DHN Melanin

Fungal strain *Mf-1* of *Mycosphaerella fijiensis* grows slowly on potato dextrose agar (PDA). The mycelium is dark green and a secreted black pigment settles at the bottom of culture dishes. In potato dextrose broth (PDB) cultures reach a logarithmic phase after 7 days of growth at 27°C, after which black mycelial pellets form and a green black pigment is secreted into the culture medium. The phenotypic characteristics of fungal strains used in this work are shown in table 1. We isolated pigments from mycelium and from the culture medium (secreted). Both pigments were insoluble in water, ethanol, acetone and chloroform. They were dissolved in 1M NaOH and precipitated with 2M HCl. The nature of the pigment was confirmed by its spectral properties. The UV spectra of the melanin isolated were compared with synthetic melanin (Sigma M8631) and exhibited a similar pattern (Figure S1). Fungal pigments absorbed strongly in the UV region and weakly at longest wavelength as previously reported [24]. The absorption spectra showed bands in the UV regions (λ = 230–300 nm) and small shoulders at 280 nm that are characteristic in all preparations studied. No peaks were present in the visible region.

**Table 1:** Comparative of phenotypic and physiological aspects of *M. fijiensis* strains used in this study.

Fungalstrain	Year and siteof collection	Hours to reachlog phase onPDB medium	Hours to secretedark pigment toPDB medium	Mycelial colour in PDA agar	Degree of Resistance to fungicides
*Mf-1*	1999, ArmeriaColima, Mexico	120	144	Dark green on the surface andblack in bottom	Higher Sensitivity to Carbendazim,Propiconazole, Azoxystrobin,Mancozeb, Chlorothalonil.
*102*	2007, Coahuayana,Michoacan, Mexico	84	204	White-pink on the surface and whitewith small black spots in bottom	Higher resistance to all fungicidesmentioned above.

The identity of single-ascospores isolates was confirmed by: the morphology of the mycelium and PCR techniques using the specific primers (ACTR/MFactF) reported previously to amplify *M. fijiensis* β-tubulin [57].

The infrared (IR) spectra of both pigments were also characteristic of fungal melanin. For melanin extracted from mycelium, the spectra displayed broad absorption bands at 3700–3000 cm^−1^ with a defined peak at 3433 cm^−1^, which corresponds to hydrogen bound groups OH and NH. The small peaks at 2953 cm^−1^ and 2853 cm^−1^ may result from aliphatic groups CH_2_ and CH_3_ stretching. Clear peaks appeared at 1711 cm^−1^ and 1628 cm^−1^ that correspond to the oscillations of C-O groups from acids, esters, ketones. The peak at 1244 cm^−1^ corresponds to C-OH stretching or angular deformation of O-H. The absorption peak at 1026 cm^−1^ is attributed to the aromatic ring C-H. The IR spectra did not show an absorption band at 1320–1390 cm^–1^ or one peak at 1540 cm^−1^ that would indicate nitrogen content (C-N bending or N-H bending respectively) as DOPA melanins (Figure S2). For secreted melanin, the IR spectrum display the same peaks at 3433, 2920, 2852, 1707, 1626, 1384, 1241 and 1026 cm^−1^ as mycelium melanin, but a peak at 1582 cm^−1^ corresponding to oscillations of the C = C bonds in a condensed aromatic system appeared distinctly in the spectra of two preparations. However, this difference does not affect the interpretation that both are considered melanin pigments lacking nitrogen in its structure.

To completely rule out the presence of nitrogen atom in the structure of both pigments, an elemental composition analysis was conducted. The elemental analysis provided C:H:N:S composition percentage for mycelium melanin was 46.68% 4.96%, 2.33% and 0.09% and 43.88%: 10.27%: 0.34:% 0.08% for melanin secreted (Table S1). The fungi that produce DOPA-melanin or fungal humic acid-type melanins, have a nitrogen content from 5% to 10% (synthetic DOPA-melanin content around 6%) [9], [25], [26]. The precursor and intermediates of 1,8-DHN synthesis: 1,3,6,8-THN, scytalone, 1,3,6, THN and vermelone does not contain nitrogen [12]. The nitrogen that normally appears in the analysis is from melanin complexed with cell wall proteins, and that protein is the source of nitrogen. The nitrogen content for secreted melanin was lower (0.34%) than melanin extracted from cell wall (2.33%), this is a further proof that both melanin types studied are derived from 1,8-DHN and that there are residues of proteins in the pigment.

**Figure 1:**
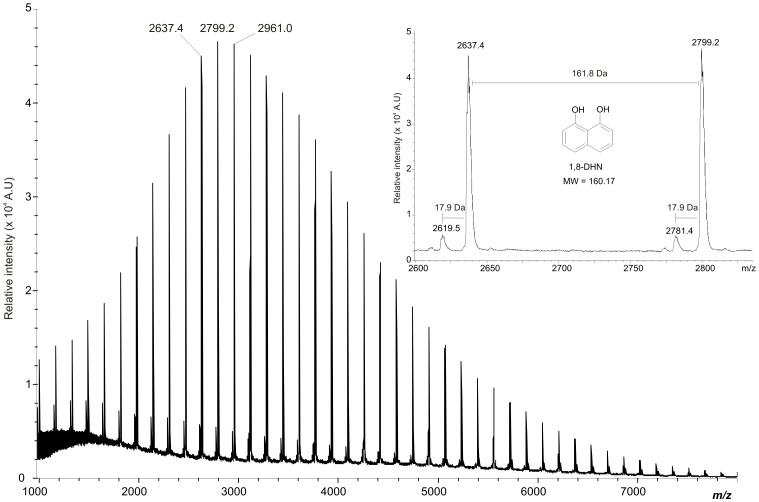
Linear-MALDI-TOF mass spectrum of 1,8-DHN-melanin extracted from *M. fijiensis*. Linear matrix-assisted laser desorption/ionization mass spectrum of DHN-melanin extracted from *M. fijiensis* (*Mf-1* strain) mycelium with NaOH 6M and precipitated with 6 M HCl during 3 hours at boiling temperature and then purified by washing in different solvents and freeze dried. Inset: Linear MALDI-TOF mass spectrum at 2600–2900 *m/z* of 1,8-DHN-melanin extracted from *M. fijiensis* (*Mf-1* strain) showing well-defined spacings of 161.8 Da between peaks.

To evaluate whether melanin of *M. fijiensis* is a DHN type, we made a structural analysis using Linear-MALDI-TOF mass spectrometry. As shown in figure 1, melanin from mycelium had molecular mass values not exceeding 8000 Da. It was further noted that a series of peaks were well separated with well-defined spacings of 161.8 Da, which is close to the theoretical mass of DHN (160.17 MW). This mass difference (Δm) of 1.6 Da could be due to MALDI-TOF low-resolution mass spectrum obtained. According to the molecular mass observed we could calculate that the pigment obtained after an extraction and purification process, is comprised 50 units of 1,8-DHN. It is also noteworthy that other minor peaks of 18 Da are present throughout the spectrum. These probably result from the dehydration process of the melanin polymer.

### The Use of 1,8-DHN-melanin Inhibitors and Identification of Related Pentaketides Confirms the Nature of the Pigment of *M. fijiensis*

**Figure 2:**
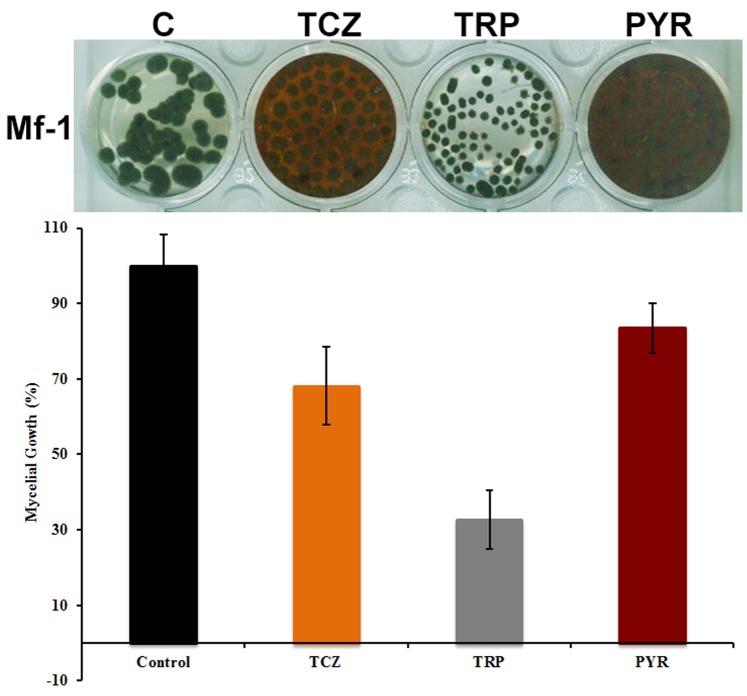
Influence of inhibitors of DHN-melanin synthesis and tropolone affects partially mycelial growth of *M. fijiensis*. Seven days-old cultures on PDB amended with 50 μg/ml of tricyclazole (TCZ), tropolone (TRP) and pyroquilon (PYR). Mycelial growth are expressed in percentage and compared to control (C). The culture media showing reddish brown color due to accumulation intermediaries product of melanin inhibition. The experiment was conducted in the *Mf-1* strain. Results are reported as the mean ± SD of 3 independent experiments.

The 1,8-DHN biosynthesis pathway was elucidated by characterizing autooxidation products, which accumulate in liquid cultures blocked in melanin biosynthesis by 50 ppm of tricyclazole and pyroquilon, two classic inhibitors that interfere with the dehydrogenation of 1,3,6,8 tetrahydroxynaphthalene (1,3,6,8-THN) to scytalone (S) and 1,3,8 trihydroxynaphthalene (1,3,8-THN) to vermelone (V) [27], [28]. The mycelial mat changed from black to reddish-brown colour and a typically reddish pigment accumulated in the culture media after 7 days culture, indicating that melanin biosynthesis was inhibited (Figure 2). Tricyclazole inhibited mycelial growth almost double compared with the pyroquilon (16.6% vs 31.8%). On the other hand, the addition of 50 ppm of tropolone (a DOPA-melanin inhibitor) to the culture medium did not cause any change in the coloration of mycelia, and did not result in secretion of soluble pigments into the culture medium as with pyroquilon and tricyclazole. Tropolone inhibits the growth of the fungus up to 67% compared with the control culture. These results support that melanin synthesized by *M. fijiensis* is a DHN-melanin.

**Figure 3:**
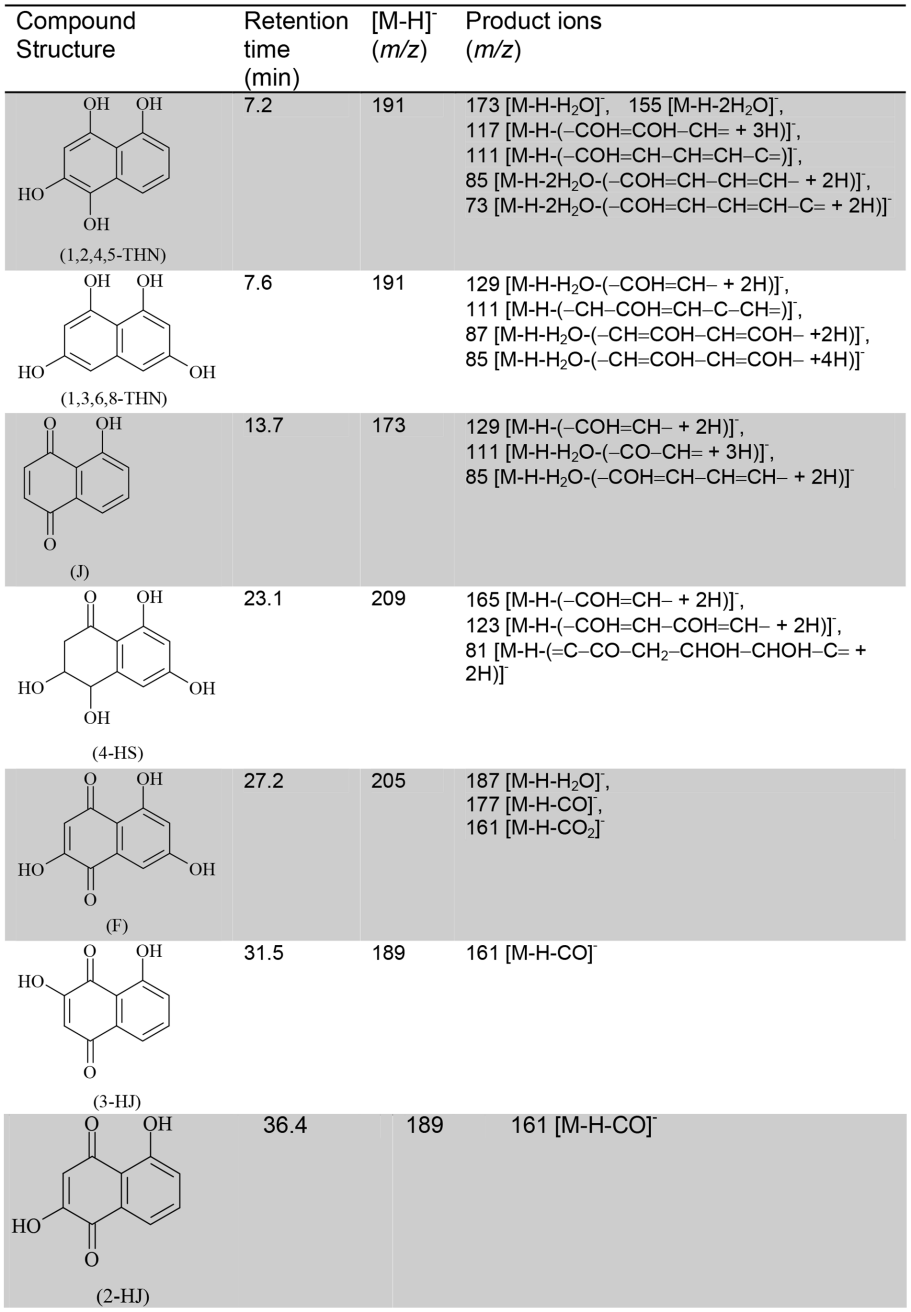
ESI-HPLC-MS/MS analysis of pentaketide metabolites accumulated in tricyclazole and pyroquilon amended culture of *M. fijiensis*. Compound names: 1,2,4,5 tetra-hydroxynaphthalene (1,2,4,5-THN), 1,3,6,8 tetra- hydroxynaphthalene (1,3,6,8 THN), Juglone (J), 4-Hydroxyscytalone (4HS), Flaviolin (F), 3-Hydroxyjuglone (3HJ), 2-Hydroxyjuglone (2HJ). The elemental compositions HPLC-ESI-MS/MS was determined in negative ion mode. [M-H]^-^ (m/z) is indicative of precursor ion. Product ions is the mass spectral fragmentation of the [M-H]^-^ produced after collision. The same compounds was found in cultures amended with Tricyclazole (TCZ) and Pyroquilon (PYR). This fungicides compound inhibits the reduction of 1,3,6,8-THN and 1,3,8-THN to scytalone and vermelone, respectively. Its strongest inhibitory effect is on the reduction of 1,3,8-THN. This results in the accumulation of F, 2-HJ, and their related shunt products. 1,2,4,5-tetrahydroxynaphthalene (1,2,4,5-THN) is an unstable metabolite identified by the method used in this work.

The ESI-HPLC-MS/MS analysis of 7 day-old PDB cultures containing tricyclazole and pyroquilon showed a complex mixture of compounds (Figure S4). In figure 3 we show the retention times, *m/z* of precursor ions, and *m/z* of product ions of the metabolites identified as: 1,3,6,8-THN (*m/z* 191), 1,2,4,5-tetrahydroxynaphathalene (1,2,4,5-THN, *m/z* 191) and typical metabolites including flaviolin (F, *m/z* 205), juglone (J, *m/z* 173), 2-hydroxyjuglone (2-HJ, *m/z* 189), 3-hydroxyjuglone (3-HJ, *m/z* 189) and 4 hydroxyscytalone (4-HS, *m/z* 209). This result confirms that tricyclazole and pyroquilon blocked the 1,8-DHN-melanin pathway. 1,3,8-trihydroxynaphthalene (1,3,8-THN) was not found, suggesting that it is rapidly autoxidized to 2-HJ. Other molecules were not identified, but were likely pentaketides since fragmentation products have similar masses characteristics of those compounds (data not show).

This is the first study where intermediary compounds of melanin synthesis were identified directly in cell-free supernatants without extracting with organic solvents. The analyses were performed in three separate assays using different batches of *Mf-1* cultures. Future studies will be focused on the elucidation of all structures accumulated by melanin inhibitors in *M. fijiensis*.

### Melanins Isolated from *M. fijiensis* Vary in Size and Structural Morphology

**Figure 4:**
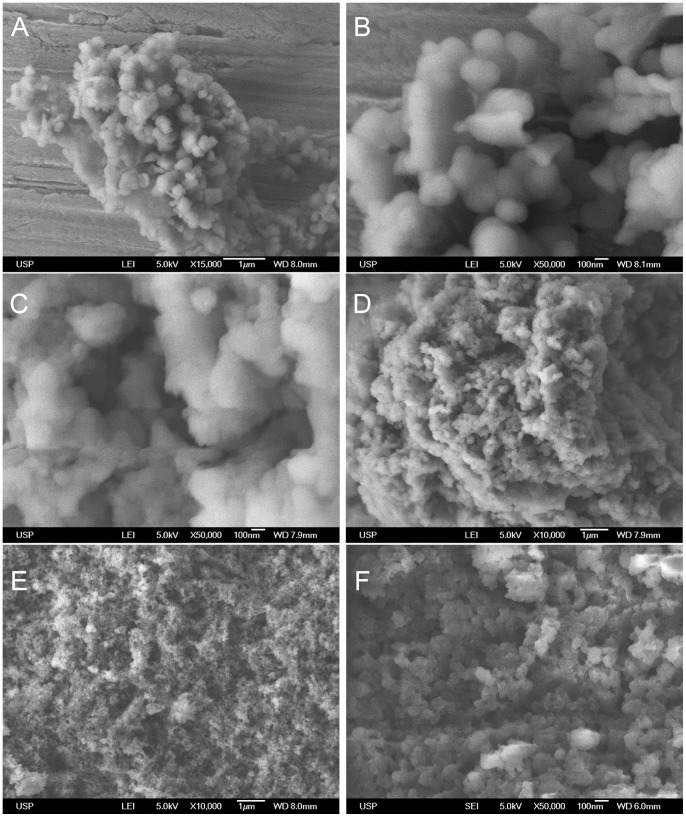
Ultrastructural morphologies of fungal melanin pigments isolated from mycelium and secreted to culture media. **A–B** are SEM images of synthetic melanin obtained by tyrosine oxidation with H_2_O_2_ (Sigma M8136); **C–D** SEM images of Melanin extracted from mycelium, this pigment displays spherical granules tightly aggregated with an average size diameter of 100–300 nm; **E–F** Melanin extracted from culture media display amorphous material without definable structure composed by tightly packed of various sizes. *M. fijiensis* pigments were obtained using NaOH extraction, acid precipitation and solvent purification methods. The scale bars are showed in each image.

It has been reported that the structural morphology and specific arrangements that melanin pigments adopt affect photoreactivity, so that unaggregated oligomers have phototoxic effects and their aggregation mitigates such processes [29]. This is the framework for understanding contrasting antioxidant and pro-oxidant roles exhibited by melanins. Any changes in structure could result in increased oxidative stress [30]. We consider it important to know their structures, as well as differences between both pigments isolated from *M. fijiensis*. Both pigments were obtained using acid precipitation, solvents extraction and freeze-drying. Figure 4 (panel A–B) shows the ultra-structural characteristics of synthetic melanin. Uniform granule bodies within a narrow size range are present and smaller granules are not observed. In the panel C–D, the melanin extracted from mycelium revealed considerable variation in particle size, with pigment existing as large clusters of spherical granules tightly aggregated with an average diameter of 100–300 nm. At a higher magnification the morphology of melanin from mycelium is quite similar to other microbial melanins reported. Panel E–F shows the structure of the secreted pigment. This is visible on the 10 μm scale as an amorphous material without definable structure, exhibiting a granular irregular surface within an area greater than 20 μm. Increased magnification, shows varying degrees of surface porosity, suggesting that this structure is also composed of tightly packed granules of various sizes. White deposits are suggested to be NaOH residues. The image with higher magnification (50,000) reveals the inside of the aggregated structure and clearly shows that the surfaces have a multi-sized stacking of particles, that appear to be comprised of granules of 20–30 nm in diameter.

### Singlet Molecular Oxygen [O_2_ (^1^Δ_g_)] is Generated by *M. fijiensis* Melanin, Melanin Intermediaries and Whole Mycelium

Singlet oxygen O_2_ (^1^Δ_g_) is the lowest electronic excited state of molecular oxygen in the ground-state [O_2_ (^3^∑_g−_)]. Production of O_2_ (^1^Δ_g_) in a biological environment can lead to oxidation of cellular constituents and cell death. A common and convenient way to produce O_2_ (^1^Δ_g_) is photosensitization in the presence of sensitizer. In this process, O_2_ (^1^Δ_g_) is generated by irradiation with UV or visible light. In this case, light absorbed by a molecule inherent or added to the system (sensitizer) will generate an excited electronic state of the sensitizer that can transfer its energy to O_2_ (^3^∑_g−_) and produce O_2_ (^1^Δ_g_).

**Figure 5:**
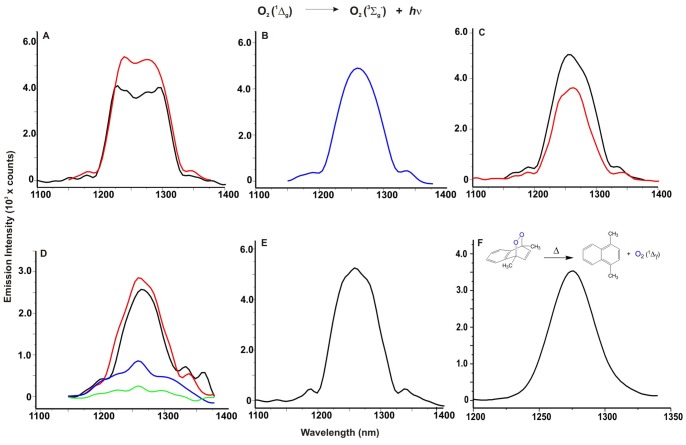
Generation of ^1^O_2_ (^1^Δ_g_) by DHN-melanin, polyketides intermediaries and living whole mycelium of *M. fijiensis*. A representative monomol light emission spectra of ^1^O_2_ at 1270 nm obtained after pulsed laser excitation at 532 nm. (**A**) Melanin pigments adjusted to 0.6 Abs in 0.1 M NaOD. Red line corresponds to secreted melanin and black line is from mycelium melanin. The phosphorescence intensity of secreted melanin was higher than mycelium melanin. (**B**) phosphorescence emission of synthetic melanin (M8136), (**C**) Reddish-brown pigment composed by polyketides and naphtoquinones mixture, was dissolved in D_2_O at Abs 0.6. Black line shows the phosphorescence of culture amended with tricyclazole and red line shows the phosphorescence of pyroquilon inhibitor. (**D**) Active whole mycelium grown in potato-dextrose culture medium. 250 mg of mycelium was used for each experiments. A black and red line corresponds to mycelium from green-black *Mf-1* strain cultured in liquid and agar media respectively. Blue and Green lines are emission spectra of albino*102* strain cultured in liquid and agar media. (**E**) Singlet oxygen production of 1,2 DHN used as standard dissolved in acetonitrile; (**F**) Emission spectra of dimethylnaphthalene endoperoxide.

The formation of O_2_ (^1^Δ_g_) from melanin pigments isolated from *M. fijiensis* irradiated by Nd:YAG laser at 532 nm is demonstrated by measurements of the monomol light emission in the near-infrared (NIR) region at 1270 nm. The monomol light emission spectra of O_2_ (^1^Δ_g_) has a maximum intensity at 1270 nm with an intensity of emission at 1270 nm that is greater for secreted melanin than melanin in mycelium (Figure 5A). This result may be due to differences in molecular size and aggregation state as shown in figure 4. The bandwidth probably is due to the polymeric nature and dynamics of formation and inactivation of O_2_ (^1^Δ_g_) within the same structure of photosensitized melanin. The O_2_ (^1^Δ_g_) spectra of the melanin standard (Figure 5B) have the same emission pattern in the NIR region as fungal melanin, and the monomol light emission spectrum of O_2_ (^1^Δ_g_) shown is consistent with reported spectra from the photosensitized hair eumelanin [6].

The addition of tricyclazole or pyroquilon to the fungal culture causes the accumulation of compounds such as naphthoquinones and hydroxynaphthalenes (Figure 3 and Figure S3). Both irradiated supernatant fractions generated O_2_ (^1^Δ_g_), however the tricyclazole supernatant (black line) had a higher emission intensity than the pyroquilon supernatant (red line) (Figure 5C). It has been reported that photoexcitation with laser flash photolysis of 1,4 naphthoquinones leads to efficient intersystem crossing to the triplet excited state which is an efficient photosensitizer for O_2_ (^1^Δ_g_) production [31], [32]. Also, It has been reported that the application of tricyclazole sprayed onto banana leaves infected with *M. fijiensis* showed very pronounced necrosis within a few days of treatment by accumulation of juglone, flaviolin, 2-HJ and 2,4,8-trihydroxytetralone [33]. Our results suggest that naphthoquinone and hydroxynaphthalene intermediaries, accumulated as by-products in banana plant tissues, are toxic to plant cells through generation of reactive O_2_ (^1^Δ_g_).

The O_2_ (^1^Δ_g_) monomol light emission from a mycelial mat of two *M. fijiensis* strains are shown in Figure 5D. We used mycelial mat of *Mf-1* (green darkness) strain that contains 8.05 μg of melanin/mg of wet mycelium and the strain *102* (albino) containing 1.98 μg/mg. Both strains show a clear signal of phosphorescence after irradiation. However the *Mf-1* strain has 3 times higher emission than strain *102*. No differences are observed in phosphorescence emission when using mycelium collected from liquid culture and the scrapped mycelium collected from an agar culture after incubation for 7 days. This is the first direct spectral evidence for ^1^O_2_ production by mycelial mats of *M. fijiensis*.

We used iso-absorptive solutions (abs = 0.6) of 1,2-DHN, 1,6-DHN, and 1,7-DHN as standards for the laser irradiation. All DHN that was probed generated O_2_ (^1^Δ_g_). Figure 5E shows the monomol light emission spectra of O_2_ (^1^Δ_g_) produced by 1,2-DHN laser irradiation. Finally in panel 5F, we show emission spectra of dimethylnaphthalene endoperoxide a clean source of singlet molecular oxygen used in this work for equipment calibration.

### Melanin is Accumulated in Necrotized Tissues of Banana Leaves Infected with Black Sigatoka Fungus

**Figure 6:**
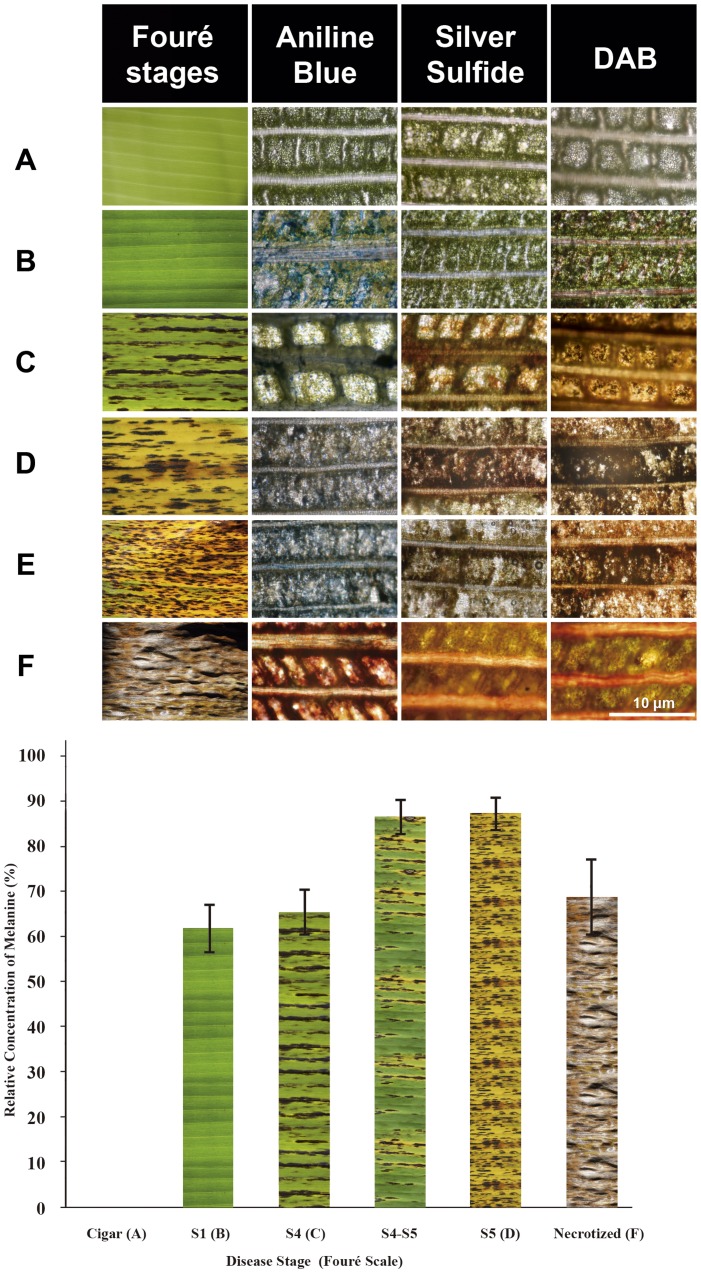
Fungal melanin content is related with necrotic damage in banana leaf naturally infected. Fouré stages of black Sigatoka disease in banana leaves naturally infected. Ten months old Giant dwarf (Cavendish) plants were collected from commercial orchard in Jalisco, Mexico. Sigatoka disease symptoms (upper panel). (**A**) Cigar leaf and (**B**) Stage 1. (**C**) Stage 4, (**D**) S5 stage, (**E**) Advanced stage 5 and (**F**) advanced necrotized stage. Scale bar = 10 μm. Relative area covered by melanin (lower panel). The values were normalized to capote leaf (used as control) that shown fungal infection. Results were obtained from at least five leaves analyzed for each stage. One representative analysis is shown. S4–S5 and S5*, showing lesions characteristic of the two stages. In the Cu-Sulfide-Silver stain, copper is bound to melanin followed by formation of copper sulfide at melanin sites, which are amplified into strong black stain using a silver enhacement step. Detection of H_2_O_2_ was performed by an endogenous peroxidase-dependent in situ histochemical staining procedure using DAB [58]. DAB polymerizes locally as soon as it comes into contact with H_2_O_2_ giving a reddish-brown polymer. In the aniline-blue staining, aniline blue reacts with beta-glucans contents in fungal hyphae.

Given these results, we next considered the hypothesis that mycelial and secreted melanins of *M. fijiensis* enhance the virulence by O_2_ (^1^Δ_g_) generation after a visible light pulse in infected leaves. This would implicate melanin as a photosensitizer toxin causing development of necrotic lesions. To evaluate our hypothesis, leaves of ten eight-months-old banana plants with high incidence of black Sigatoka were obtained from a commercial orchard and carefully tended to analyze the presence of melanin in leaf tissues during the progress of natural infection and the development of necrotic lesions. In order to show the presence of melanin in tissues by silver sulfide staining (Figure 6, below) and its importance in causing leaf tissue damage, we separated the results of semi-quantitative analysis of the covered area with the fungus (based on incorporation of aniline blue into fungal hyphae) and the presence of H_2_O_2_ in the plant tissue (detected as a reddish-brown color) in each of the stages of the disease, these are shown in Figure S5.

Figure 6A shows a fragment of unfolding “cigar” or “candela” leaf that was used as control for melanin stain. The fungus did not infect the cigar leaf and no melanin and H_2_O_2_ were detected in tissues (Figure S5) Figure 6B shows a leaf fragment in infection stage 1 (S1) according to the Fouré scale [34]. The leaves at this stage tend to lose the bright green to pale green color, without manifestation of black Sigatoka lesions. At the light microscopic level, the aniline blue stain shows a greater staining of fungal hyphae (in deep blue) distributed among the cells of palisade layer and intercellular spaces of leaf mesophyll; with hyphae covering approximately 27% of leaf area analyzed (Figure S5). At this stage of infection, melanin is not detected in leaf tissue or in areas where hyphae are located, however H_2_O_2_ was detected in 7.4% of area analyzed. In Figure 6C a banana leaf fragment has wider brown streaks at the S4 stage (also called “the first spot stage”) with discoloration and loss of vigor. Microscopically, we observed fungal structures, including asexual conidia, on mesophyll cells. No significant difference was found in the areas covered by the fungus compared to previous stages. In addition, melanin covered 20.43% of the leaf area. However DAB staining shows 40% of the area with H_2_O_2_ presence in the intercellular spaces and light brown deposits in the cells of the palisades layer. It is difficult to distinguish whether the fungus or the plant is responsible for accumulation of hydrogen peroxide in this site. In the transition from stage S4 to S5 no change in the area covered by the fungus was observed, but the H_2_O_2_ level approached 50%. In the transition from stage S4 to S5 melanin increased by 5%. Symptoms of S5 or “second spot stage” in banana leaf are shown in the Figure 6D. This stage is characterized by many black spots with yellow halos. At this stage the fungus has penetrated the cells causing irreversible structural changes in mesophyll cells. Intracellular penetration damages plant cell membranes and increases nutrient leakage into intercellular spaces. Although the fungal area diminished 5%, the number of reproductive structures (pseudothecia) increased. Melanin reaches the maximum value 28.6% in this stage of disease progression. The amount of H_2_O_2_ at this stage is greater than in previous infection stages. It is estimated that this stage occurs after 50–60 days post-penetration of stomata. This finding suggests that the amount of fungal melanin deposited within foliar tissues is directly related to mesophyll cell death and necrosis. Figure 6E shows a leaf in advanced S5* stage. Here is an increase in chlorotic regions, and the black streaks are larger. There are decreases in mycelium content of plant tissues and plant cell morphologies are altered. A decrease in the melanin content and H_2_O_2_ production were observed. In Stage 6 (Figure 6F), leaves may be completely necrotized colorless and colorless with gray halos and black dots (pseudothecia) within them. Microscopically, we observed a loss of plant tissue structure and a decrease in the mycelium content up to 5% was observed.

## Discussion

In this study, we report that the pigments contained in the mycelium and secreted into the culture medium of *M. fijiensis* are melanins that absorb visible light and act as photosensitizers that can generate O_2_ (^1^Δ_g_). Based on these findings, our work suggests that melanin should be further studied as a potentially important contributor to progression of black Sigatoka disease of bananas and plantains. In this research we identified a melanin pigment from mycelium and secreted into culture media as DHN-melanin, the sole melanin-type in *M. fijiensis*. The molecular mass obtained by MALDI-TOF analysis is approximately 8000 Da and shows a molar mass distribution with peak-to-peak increments of 161.8 Da. It is possible to see other peak distributions with less extension than 8000 Da, but with the same molar mass distribution. This may be due to the harsh chemical treatment that is required to extract and purify melanin. The polymer must be hydrolyzed to small oligomeric fragments. The polymerized form would have a higher molecular mass. Fungal melanin synthesized *in vitro* using laccase and 1,8-DHN has a molecular mass greater than 60 KDa [35].

To ascertain the chemical nature of *M. fijiensis* melanin, inhibitors may be used to identify the type of melanin synthesized by a fungus. Compounds such as tricyclazole, pyroquilon, fthalide and chlobenthiazone inhibit DHN-melanin synthesis but not DOPA melanin [36]. We found by ESI-HPLC-MS/MS that the use of pyroquilon and tricyclazole increased the accumulation of typically naphthoquinone intermediates by inhibition of scytalone dehydratase such as flaviolin, juglone, 4-Hidroxyscitalone, 3-HJ and 2-HJ (Figure 3). The generation of flaviolin and 2-HJ in fungal cultures treated with tricyclazole, is usually accepted as proof of the presence of 1,3,6,8-THN and 1,3,8-THN that were involved in the synthesis of 1,8-DHN. Also the unstable intermediate 1,2,4,5-THN and the precursor of the synthesis of 1,8-DHN, the 1,3,6,8-THN was identified. We interpret the accumulation of these compounds in the culture media to be an indication of an inhibitory effect on mycelial growth (Figure 2). This hypothesis is supported by formation of intermediary compounds that may act as photosensitizers and generate O_2_ (^1^Δ_g_) (Figure 5). Decreases in the melanin accumulated in the fungal cell walls probably increase the cytotoxic effects of ^1^O_2_. This is because melanin can function as a scavenger of this ROS, reducing oxidative damage to cell membrane components, mainly lipids, as has been demonstrated for eumelanin [37], [38]. Interestingly, tropolone strongly inhibits mycelial growth compared to pyroquilon and tricyclazole. Tropolone is classified as a DOPA melanin inhibitor, however it is also used to inhibit laccase, an enzyme involved in the oxidative synthesis of DHN-melanin. Therefore, the inhibition of this enzyme could be a target for the design of new fungicides. This is especially possible because DHN-melanin is primarily produced in the fungal kingdom, particularly Ascomycota.

Melanin in biological systems is usually associated with protection from UV radiation and as free radical trap [39]. However, melanins also produce ROS upon UV illumination. This dichotomy of photoprotection and phototoxicity are linked to the polymer backbone [29]. Melanins isolated from *M. fijiensis* are structurally similar to the other microbial melanins [40], [41]. They consist of a spherical granular body arrangement in clusters with different sizes and aggregations (Figure 4). Higher magnifications of secreted and hyphal melanin revealed a substructure of spherical units that were variable in size. The size of granular aggregates was higher in the melanin obtained from the cell walls than melanin secreted into the culture medium (Figure 4). It has been reported that melanins associated with cell walls consist of large granules of 50–80 nm in diameter with multiple layers and with an amplitude similar to the diameter of the granule [42], the presence of large granules decrease the porosity and increase the absorptive properties to toxic molecules in the polymer. These structural differences between melanin pigments should be considered with respect to O_2_ (^1^Δ_g_) generation. The photochemical properties of eumelanin polymer have been considered as a consequence of aromatic rings or hydroxyl moieties; this should also apply to DHN-melanin. Figure 5 shows characteristic monomol light emission spectrum of O_2_ (^1^Δ_g_) generated by fungal melanins. The secreted melanin produces more O_2_ (^1^Δ_g_) than mycelial melanin and commercial melanin. This supports the idea that an aggregated structure prevents ROS formation because photoactive residues are less exposed. This fact has interesting biological implications because secreted melanin becomes a chemical weapon for cell destruction in order to access nutrients in the plant cell, especially in the necrotrophic phase. In contrast, aggregated melanin in the mycelium probably serves to protect fungal hyphae from O_2_ (^1^Δ_g_) damage. Despite peak shifting ±10 nm, the phosphorescence at 1270 nm is unequivocal evidence of the generation of O_2_ (^1^Δ_g_) in our assays. It is notable that the spectra of fungal melanins are wide and less defined compared with the spectra obtained from commercial melanin and the compounds accumulated during melanin inhibition (Figure 5B and 5C). Perhaps this is directly related to the structure *per se* of fungal melanins, where irradiation causes the generation of O_2_ (^1^Δ_g_), but is disabled by the same structure after a while, causing the phosphorescence peak uncertainty. Melanins have intrinsic content of quinone/semiquinone radicals, but more semiquinone radicals are reversibly photogenerated, favoring the formation of ROS [43]. Quinones and hydroxyquinones are excellent generators of O_2_ (^1^Δ_g_) but also are good quenchers after photoirradiation [44].

Singlet molecular oxygen plays a number of different and important roles in biological systems. Singlet molecular oxygen may serve as an oxidizing molecule leading to lipid peroxidation and DNA oxidative damage [45], [46], [47], [48], [49]; therefore the fact that singlet molecular oxygen can perturb and/or lead to the destruction of molecules, cells, or tissues can have adverse effects on a healthy and well-functioning organism.

Particular strains of *M. fijiensis* promote O_2_ (^1^Δ_g_) generation after irradiation. As we show in figure 5D, the mycelium of the dark strain (*Mf-1*, Figure S3) generates more ^1^O_2_ than the albino strain (*102*, Figure S3); therefore ^1^O_2_ generation by melanin becomes a condition that causes oxidative stress and host cell death. Albino strains of *M. fijiensis*, compensate for melanin deficiency producing carotenes and higher catalase activitiy (unpublished results) and can infect banana leaves, but the damage is not so severe [21]. It has been long observed that banana plants infected with black Sigatoka are less severely affected in the shade than in full sunlight, suggesting the production of light activated toxins by these fungi. Indeed, there are reports that *M. fijiensis* produce phytotoxins that will be activated by light, some of them related to melanin biosynthesis [33], [50], [51], [52], [53]. In the past some research groups have searched in *M. fijiensis* for a toxin like cercosporin, because *M. fijiensis* is closely related to *Cercospora* species, which produces cercosporin, a light-activated phytotoxin. However, this toxin has not been found in *M. fijiensis*. Cercosporin is a perilenequinone toxin produced by pathogenic species of *Cercospora*, where UV radiation leads to photosensitization and production of singlet molecular oxygen. This phenomenon has been associated with cellular death by necrosis, thus favouring the access to nutrients to support the growth of the intercellular pathogen [50].

From our point of view, the accumulation and secretion of DHN-melanin in *M. fijiensis* also known as polyketide melanin acts as a photoactivated “toxin”. To support our hypothesis that melanin is associated with leaf necrosis, we analysed leaves of the susceptible banana variety (dwarf giant, Cavendish AAA) with symptoms of black Sigatoka. Leaf fragments in different disease stages were analysed microscopically and compared with cigar leaves and leaves infected by the fungus without necrotic symptoms. The leaf at stage S1 (Figure 6B) was colonized but did not show presence of melanin. The H_2_O_2_ was localized in mesophyll cells adjacent to mycelium in leaves without apparent damage. Leaves in later stages of the disease, showed accumulation of melanin and H_2_O_2_, suggesting that the leaf tissues were under oxidative stress and this was further evidenced by destruction of mesophyll cells. The highest levels of H_2_O_2_ accumulation occured at stage S1 and in the S4–S5 transition. In necrotic leaves the area in which H_2_O_2_ was detected decreases (Figure 6F). Moreover melanin detection begins after S1 and has its maximum accumulation in S5. A statistical analysis shows an H_2_O_2_/melanin coefficient of 0.9669. However H_2_O_2_ appears at an earlier stage, suggesting that H_2_O_2_ may be secreted by the plant as part of the defence mechanism and probably acts as inducer of fungal melanin synthesis It is important to note that in fully necrotized leaves the presence of melanin was not observed, but we found fungal reproductive structures (pseudothecia), which indicate that the fungal life cycle has been completed.

All plants need light to grow and some microbes require light for pathogenesis [54]. Banana leaves are exposed to 10–14 hours daily to intense sunlight. After penetrating through the stomata, *M. fijiensis* lives in the plant as asymptomatic endophytic mycelia for a few weeks prior to symptom expression. Factors such as plant defence response or fungal biomass accumulation associated with need for nutrients may stimulate melanin synthesis. Thus when this occurs the fungus is more resistant to ROS generated by either plant or fungus. In addition, accumulated melanin probably acts as sunscreen that reduces photosynthesis, further weakening the host. Melanin-mediated singlet molecular oxygen production leads to lipid peroxidation and breakdown of a host plant’s plasma membranes. In this respect, melanin probably acts as a light-activated phytotoxin. This provides host cellular access and nutrients, since leaf colonization initially is limited to leaf intercellular spaces.

## Conclusions

O_2_ (^1^Δ_g_) generation by the DHN-melanin, provides an answer to a question about the effects of hyphal melanization in the fungus-plant host interaction formulated some years ago by Dr. Michael J. Butleŕs group [55]. DHN-melanin in the banana-*M. fijiensis* interaction functions as a deleterious molecule to the plant tissue. The mycelial melanin probably deactivates singlet molecular oxygen and acts as a fungal hyphal protector. This result provides information about hyphal melanization in fungal-plant interactions and suggests why hyphae are melanized. Our research raises new questions about the participation of ^1^O_2_ and melanin in the pathogenesis process in plants. For example: Is ^1^O_2_ produced by melanin responsible for the change to pathogen in fungal endophytes? [56], It is melanin synthesis the cause or effect of the resistance/adaptation to oxidative stress? An understanding of the function of melanin-mediated singlet molecular oxygen for this and other pigmented fungal pathogens is critical to development of a comprehensive understanding microbial pathogenesis.

## Materials and Methods

### Fungal Strains and Culture

The strains *Mf-1* and *102* of *M. fijiensis* collected in Mexico were cultured in Petri dishes containing Potato Dextrose Agar (PDA) medium (DIFCO) and the identity of single-ascospores strains was confirmed by PCR techniques using the specific primers (ACTR/MFactF) reported previously to amplify *M. fijiensis* β-tubulin [57]. The Petri dishes were maintained for 7 days in a growth chamber at 27C. After incubation pieces of mycelium (1 cm^2^) were transferred to 250 ml Erlenmeyer flasks with 50 ml of Potato Dextrose Broth (PDB) and incubated for 7 days on a rotary shaker at 150 rpm at 27°C. The mycelial mats were harvested by filtration in a Büchner funnel with Whatman filter paper (#1), washed with distilled water and frozen at −80°C until melanin extraction.

### Tricyclazole, Pyroquilon and Tropolone Inhibition Assay

Tricyclazole (5-methyl-1,2,4-triazole[3,4-b]benzothiazole), pyroquilon (1,2,5,6-tetrahydropyrrolo [3,2,1,-ij]quinolin-4-one), and tropolone (2-Hydroxy-2,4,6-cycloheptatrien-1-one) were purchased from Sigma-Aldrich (St. Louis, MO) and used at 50 ppm to study *in vivo* the effect on melanin biosynthesis and the mycelium growth effect. Both were dissolved in ethanol (10 mg/ml stock solution) and added to 50 ml of potato dextrose broth (PDB) (Difco Laboratories). To estimate fungal biomass growth, 100 mg of mycelial mat was obtained from 7-day-old cultures that were actively grown in PDB. This was put in a 12-multiwell plate with 2.5 mL of fresh PDB medium for each well and incubated for 7 days at 27°C and 150 r/min. After incubation, the mycelia were filtered through Whatman filter paper *in vacuo* to separate them from the culture. The material was dried to constant mass at 60°C and weighed. The experiments were performed in triplicate. Results are reported as the mean ± SD.

### Melanin Isolation from Mycelia and Liquid Media

#### a) Melanin extraction from Mycelia

Mycelial mats were ground with mortar and pestle and then extracted with 2M NaOH (dilution coefficient 1∶10) at 100°C for 3 h in a round bottom flask with reflux condenser. The cell extract was centrifuged at 5000 g and the supernatant was acidified with HCl concentrated until precipitation at pH 2.0. The resulting precipitate was recovered by centrifugation at 5000 rpm for 20 min and the precipitate was purified by acid hydrolysis using 6M HCl at 100C for 2 h to remove carbohydrates and proteins. The non-hydrolysable residues were collected by centrifugation at 5000 rpm for 20 min and then successively treated with ethanol and chloroform to remove lipids. The residue obtained was dried at room temperature and re-dissolved in 2M NaOH and centrifuged at 4000 g for 15 min. The supernatant was precipitated by adding 2M HCl washed five times with milliQ water, filtered and lyophilized.

#### b) Melanin extraction from Culture media

The pigment present in the mycelium free culture medium was extracted by acidification to pH 1.5 with 10M HCL and then heated to boiling with gentle stirring for 30 minutes and cooled to ambient temperature, after which a precipitate formed. This solid was filtered using a Millipore membrane of 0.45 μm and washed several times with Milli-Q water until the pH of the filtered water was 7. Afterward this solid was washed three times with 50 ml of ethanol and 50 ml of chloroform to remove proteins and lipids. The residue was redissolved in 1∶1 ethanol-water Milli-Q and lyophilized.

### Characterization of the Black Pigments Extracted from Mycelia and Culture Media

#### Ultraviolet-Visible and infrared spectroscopy

The isolated and purified melanin from mycelia and liquid media were dissolved in 1 ml of NaOH 0.1M at a final concentration of 0.5 mg/ml and its ultraviolet-visible spectrum was measured in a Shimadzu spectrophotometer UV-1650 PC (Shimadzu Scientific Instruments, Columbia, MD) at a wavelength of 200–600 nm. The infrared spectra of the pigments were recorded on an FT-IR spectrometer Bomem model MB100 (ABB Bomem Inc, Quebec, Canada) using KBr pellets obtained by pressing uniformly at 7 metric tons prepared at 1 mg of pigment sample and 100 mg of spectrometry grade KBr, over the range 4000-400 cm-1, using 4 cm^−1^ resolution.

### Elemental Analysis

Elemental analyser (Perkin Elmer Series II CHNSO/O Analyser 2400, Waltham, MA, USA) was utilized to determine the percentage content of C, H, O and N in melanin pigments.

### Scanning Electronic Microscopy

Sample preparation was done by dispersing one milligram of melanin pigments of *M. fijiensis* and commercial melanin in ethanol. The sample solution was deposited and air-dried on the reflective surface of a silicon wafer. Scanning Electron Microscopy analysis was performed on a JEOL JSM-7401F FEG (Field Emision Gun; JEOL Ltd, Tokyo).

### MALDI-TOF Mass Spectrometry Analysis

Matrix-Assisted Laser Desorption/Ionization (MALDI) analysis was performed on an Ultraflextreme Time of flight mass spectrometer (Bruker-Franzen Analytik, Bremen, Germany) in positive linear mode. Ions formed by a pulsed UV laser beam (nitrogen laser, A = 337 nm) were accelerated to 20 keV. UV laser light (energy about 30) was focused onto the sample, using a focal diameter of about 100–300 nm. Melanins were mixed with twenty microliter of 2,5 dihydroxybenzoic acid (200 mg in a 30/70/0.1 acetonitrile/water/triflouroacetic acid) solution by triplicate. One microliter was deposited on the stainless steel sample holder and air-dried before introduction to the MALDI-TOF mass spectrometer. The composite mass spectra obtained are the average of 100 laser shots taken from 10 distinct positions across the sample deposit.

### ESI-HPLC–MS/MS Analysis of Intermediate Products of DHN-Melanin Synthesis Inhibition

Two grams of mycelium were inoculated and incubated for 7 days. The pentaketide accumulated were identified by High-Performance Liquid Chromatography (Shimadzu HPLC system, Tokyo, Japan) coupled to mass spectrometry in tandem (Quattro II mass spectrometer, Micromass, Altricham, UK) using electrospray ionization source (ESI-HPLC-MS/MS). For ESI-HPLC-MS/MS analysis, 100 μl of the mycelium free supernatant (centrifuged and filtered by 0.22 μm membrane [Millipore]) was injected into a Phenomenex Gemini C18 column (250 mm 4.6 mm, 5 μm particle size) and eluted with 0.1% formic acid (A) and acetonitrile (B) using 0.6 ml/min of flow rate. From flow rate, 10% was directed for mass spectrometer. The mixture of compounds was separated by a linear gradient of 10 to 40% during 30 min, 40% during 10 min, 10% for 2 min and 10% until 45 min. The column oven temperature and UV detector were set at 21°C and 250 and 280 nm, respectively. The mass spectrometry analysis was performed using negative ion mode and the following parameters: source temperature at 100°C; de-solvation temperature at 200°C; capillary voltage of 2 kV; and sample cone voltage and extractor cone voltage at 30V and 5V respectively. The collision energy was 10 eV. The flow rates of drying and nebulizing gases were 400 and 15 L/h, respectively.

### Laser Flash Photolysis and Singlet Oxygen Generation from Isolated Melanins and Whole Mycelia

Singlet oxygen measurements were performed in an Edinburgh F900 instrument (Edinburgh, UK) equipped with a Continuum Surelite III laser (5 ns duration, 10 pulses/s, 7.8 mJ/pulse), cuvette holder, silicon filter, monochromator and liquid nitrogen–cooled NIR PMT (R5509) from Hamamatsu (Hamamatsu, Co., Bridgewater, NJ, USA) and a fast multiscaler analyser card with 5 ns/channel (MSA-300; Becker & Hickl, Berlin, Germany). The emission spectra of singlet molecular oxygen monomolecular light emission were obtained by measuring emission intensities from 1099 to 1390 nm with 5 nm steps and automatically constructed by the instrument software by acquiring decays at various wavelengths and plotting the maximum emission intensity in each wavelength. Melanins (5.0 mg) isolated from fungal cell wall, culture media and melanin standard from sigma (M8631) were suspended in 5 ml of 0.1 M NaOH/D_2_O. Melanin solutions were adjusted to 0.6 units of absorbance at 532 nm and the NIR spectral were performed after two hours maximum of samples preparation. Two hundred and fifty milligrams of fungal mycelia from 7 days of incubation were suspended in 2.5 ml of D_2_O immediately after collecting directly from culture media (liquid and agar) and carefully placed inside a fluorescence quartz cuvette with a pipette and were irradiated by the Nd:YAG laser.

### Laser Flash Photolysis of Inhibitory Products of Melanin Synthesis

The reddish-brown pigmented culture was filtered through gauze, centrifuged at 5000 rpm for 20 minutes and re-filtering using a 0.22 μm Millipore membrane to remove all mycelium. The filtrate was stored at 4°C until use. The filtrated was adjusted to 0.6 units of abs at 532 nm and irradiated by Nd:YAG laser. At the same conditions of melanin described above. To make the standard of 1,2 DHN was dissolved 5 mg of DHN in 5 ml of acetonitrile and adjusted to 0.6 absorbance units.

### Melanin and H_2_O_2_ Stains in Naturally Infected Banana Leaf with Sigatoka

#### Plant Material

Naturally Sigatoka infected banana leaves were obtained from Giant Dwarf Cavendish plants (*Musa acuminata* AAA) from commercial orchard “UVA” located in the Municipality of Cihuatlán, Jalisco (19° 12 ′52.76′′N and 108° 34′ 25.62′′ W) at an altitude of 18 masl. Five leaf samples were taken representing the one of the six disease stages described by Fouré [35], plus samples from cigar leaf and first leaf after cigar leaf completely healthy in appearance. These were placed in paper bags 80×50 cm to be transported to the laboratory in a cooler keeping at 4°C in order to prevent the development of histological changes. Cut leaf tissue with appropriate lesions into 5 mm^2^-long pieces were made manually using a new scalpel blade # 5 and then were processed using three different stains (aniline blue/lactic acid, DAB staining and sulphide-silver staining).

### Aniline Blue/Lactic Acid Staining

All leaf fragments (4 replicates per each Foure Stage) were incubated by 3–5 min in 1% aniline blue/lactic acid and then were two times washed by 30 min in distilled water. Leaves were mounted directly on slides and examining using bright field microscopy at 40X and 100X. Observations were made using a light microscope Carl Zeiss Axiolab (Germany). Fungal mycelium stained blue deep and appear growing around and about the mesophyll cells.

### DAB Staining

Detection of H_2_O_2_ by 3,3-diaminobenzidine (DAB staining was performed as described previously [58]. Tissue pieces from banana leaves were incubated in a solution of DAB (1 mg/ml), 0.05 M Tris-HCl (pH 3.8) by 5 h in darkness. After this time leaves were washed three times in distilled water by 10 min to remove traces of DAB and subsequently investigated using a bright-field microscope. The presence of hydrogen peroxide was revealed by the formation of brown insoluble precipitate in the tissue.

### Melanin Staining (Sulfide-Silver Staining)

To melanin staining we follow the procedure proposed by Butler et al. [59]. Banana leaves were pretreated for 2 hours in a 10 mM copper sulfate solution in distilled water at room temperature. Subsequently rinsed in 10 ml distilled water for 1 minute, and then were placed in a 1% sodium sulfide keeping at 45–50°C for an hour in dark and after washing in distilled water. Cuts were dried for 10 min and developed in 30 ml solution containing silver lactate 22 mg and 170 mg of hydroquinone in a citrate buffer (0.1 M, pH 3.7) solution for 30 min to 1 hour at 26°C. Treated leaves were mounted directly on slides and examining using bright field microscopy at 40X and 100X. The copper-sulfide-silver procedure gives solid staining over tissue surface, which is purple-black.

All pictures were taken in one session and the image acquisitions were take using light microscope Carl Zeiss Axiolab equipped with a Canon 40D camera adapted to microscope connected to a computer (Imac; Apple).

### Data Analysis

Accumulated melanin and H_2_O_2_ data were analyzed using STATGRAPHICS Centurión XVI v. 16.1.02 software. Data are given as mean ± SD. One-way Analysis of Variance (ANOVA) with significance level set at α = 0.05 was used to determinate the statistical difference between Fouré Stages. Simple and Multiple Regresion was used to determinate the correlation among melanin, DAB Stain (H_2_O_2_) and the Fouré Stage.

## Supporting Information

Figure S1 **UV and visible spectra of melanin pigment isolated from** ***Mycosphaerella fijiensis Mf-1*** **strain.** Extracted from mycelium (**A**) and from culture medium (**B**) in comparison with synthetic melanin (**C**). Freshly melanins solutions at final concentration of 40 μg/ml in 0.1 M NaOH (for A and B) and 10 μg/ml in 0.1 M NaOH (C) were prepared for UV analysis.
(DOCX)

Figure S2 **Infrared spectra of melanin extracted from mycelia (A) and secreted to the culture medium (B).** The infrared spectra of the pigments were using KBr pellets obtained by pressing uniformly at 7 metric tons prepared at 1 mg of pigment sample and 100 mg of spectrometry grade KBr, over the range 4000–400 cm^−1^, using 4 cm^−1^ resolution.
(DOCX)

Figure S3 **Morphological aspects of** ***M. fijiensis*** **strains** ***Mf-1*** **and** ***102*** **used in this study.** The fungal strains were cultivated on Potato Dextrose (PDA) and were incubated by 7 days at 27°C.
(DOCX)

Figure S4 **ESI-HPLC-MS/MS analysis of pentaketide metabolites accumulated in tricyclazole and pyroquilon amended culture of** ***M. fijiensis*** **.** UV chromatogram at 250 nm (A). Product ions mass spectrum of 1,2,4,5-THN with *m/z* 191 (B); 1,3,6,8-THN with *m/z* 191 (C); J with *m/z* 173 (D); 4-HS with *m/z* 209 (E); F with *m/z* 205 (F); 3-HJ with *m/z* 189 (G) and 2-HJ with *m/z* 189 (H).
(DOCX)

Figure S5 **Semi-quantitative analysis of the covered area with the fungus (based on incorporation of aniline blue into fungal hyphae) and the presence of H_2_O_2_ in the plant tissue (detected as a reddish-brown color) in each of the stages of the disease.** The values were normalized to “capote” leaf (used as control) that shown fungal infection. Results were obtained from at least five leaves analyzed for each stage. Data are given as mean ± SD. One-way Analysis of Variance (ANOVA) with significance level set at α = 0.05 was used to determinate the statistical difference between Fouré Stages.
(DOCX)

Table S1 **Elemental analysis of isolated melanins of** ***Mycosphaerella*** **fijiensis.**
(DOCX)
