# Supplementary material for: Singlet Molecular Oxygen Generation by Light-Activated DHN-Melanin of the Fungal Pathogen Mycosphaerella fijiensis in Black Sigatoka Disease of Bananas
Source: PLoS One. 2014 Mar 19;9(3):e91616. doi: 10.1371/journal.pone.0091616 (PMC3960117; doi:10.1371/journal.pone.0091616)
Supplement: Figure S1 — UV and visible spectra of melanin pigment isolated from Mycosphaerella fijiensis Mf-1 strain. Extracted from mycelium (A) and from culture medium (B) in comparison with synthetic melanin (C). Freshly melanins solutions at final concentration of 40 μg/ml in 0.1 M NaOH (for A and B) and 10 μg/ml in 0.1 M NaOH (C) were prepared for UV analysis. (DOCX) [file pone.0091616.s001.docx]

**Figure S1.** UV and visible spectra of melanin pigment isolated from *Mycosphaerella fijiensis* *Mf-1* strain. Extracted from mycelium (**A**) and from culture medium (**B**) in comparison with synthetic melanin (**C**). Freshly melanins solutions at final concentration of 40 μg/ml in 0.1 M NaOH (for A and B) and 10 μg/ml in 0.1 M NaOH (C) were prepared for UV analysis.
