# Supplementary material for: Singlet Molecular Oxygen Generation by Light-Activated DHN-Melanin of the Fungal Pathogen Mycosphaerella fijiensis in Black Sigatoka Disease of Bananas
Source: PLoS One. 2014 Mar 19;9(3):e91616. doi: 10.1371/journal.pone.0091616 (PMC3960117; doi:10.1371/journal.pone.0091616)
Supplement: Figure S2 — Infrared spectra of melanin extracted from mycelia (A) and secreted to the culture medium (B). The infrared spectra of the pigments were using KBr pellets obtained by pressing uniformly at 7 metric tons prepared at 1 mg of pigment sample and 100 mg of spectrometry grade KBr, over the range 4000–400 cm−1, using 4 cm−1 resolution. (DOCX) [file pone.0091616.s002.docx]

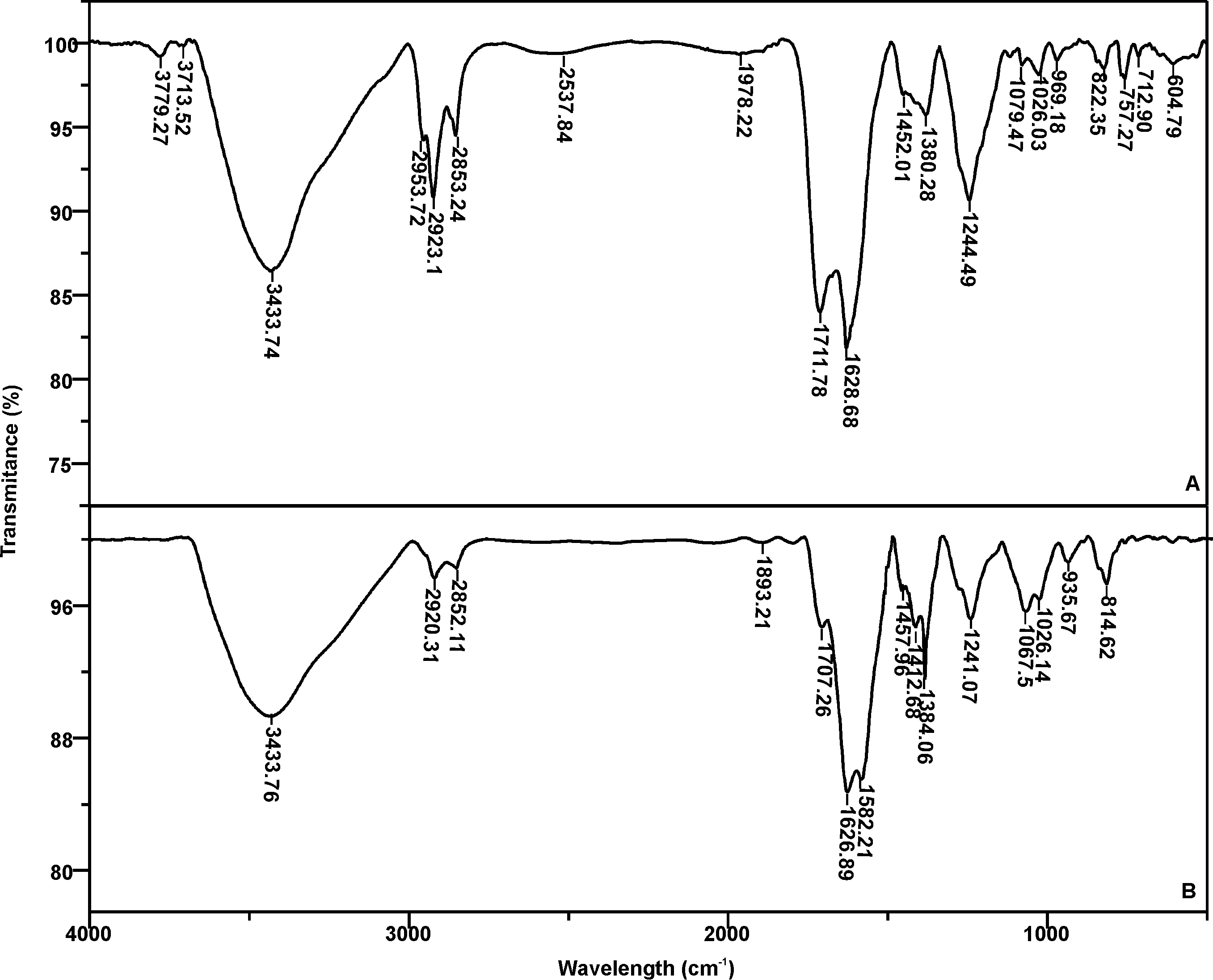


**Figure S2.** Infrared spectra of melanin extracted from mycelia (A) and secreted to the culture medium (B). The infrared spectra of the pigments were using KBr pellets obtained by pressing uniformly at 7 metric tons prepared at 1mg of pigment sample and 100 mg of spectrometry grade KBr, over the range 4000-400 cm-1, using 4 cm^-1^ resolution.
