# Supplementary material for: Singlet Molecular Oxygen Generation by Light-Activated DHN-Melanin of the Fungal Pathogen Mycosphaerella fijiensis in Black Sigatoka Disease of Bananas
Source: PLoS One. 2014 Mar 19;9(3):e91616. doi: 10.1371/journal.pone.0091616 (PMC3960117; doi:10.1371/journal.pone.0091616)
Supplement: Figure S3 — Morphological aspects of M. fijiensis strains Mf-1 and 102 used in this study. The fungal strains were cultivated on Potato Dextrose (PDA) and were incubated by 7 days at 27°C. (DOCX) [file pone.0091616.s003.docx]

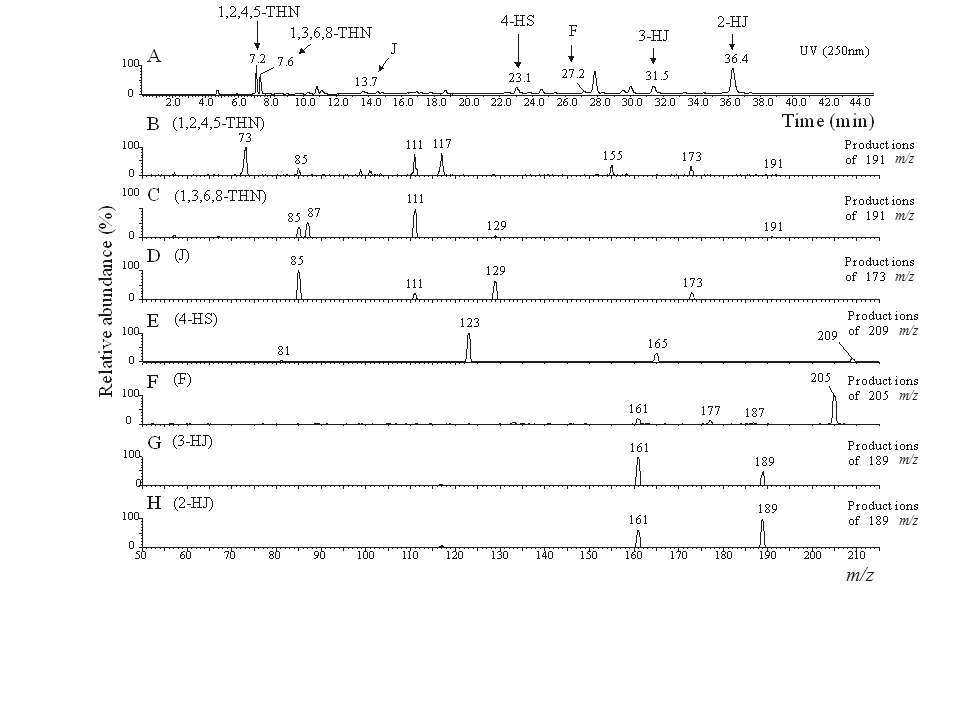


**Figure S3**. ESI-HPLC-MS/MS analysis of pentaketide metabolites accumulated in tricyclazole and pyroquilon amended culture of *M. fijiensis*. UV chromatogram at 250 nm (A). Product ions mass spectrum of 1,2,4,5-THN with *m/z* 191 (B); 1,3,6,8-THN with *m/z* 191 (C); J with *m/z* 173 (D); 4-HS with *m/z* 209 (E); F with *m/z* 205 (F); 3-HJ with *m/z* 189 (G) and 2-HJ with *m/z* 189 (H).
