# Supplementary material for: Singlet Molecular Oxygen Generation by Light-Activated DHN-Melanin of the Fungal Pathogen Mycosphaerella fijiensis in Black Sigatoka Disease of Bananas
Source: PLoS One. 2014 Mar 19;9(3):e91616. doi: 10.1371/journal.pone.0091616 (PMC3960117; doi:10.1371/journal.pone.0091616)
Supplement: Figure S4 — ESI-HPLC-MS/MS analysis of pentaketide metabolites accumulated in tricyclazole and pyroquilon amended culture of M. fijiensis . UV chromatogram at 250 nm (A). Product ions mass spectrum of 1,2,4,5-THN with m/z 191 (B); 1,3,6,8-THN with m/z 191 (C); J with m/z 173 (D); 4-HS with m/z 209 (E); F with m/z 205 (F); 3-HJ with m/z 189 (G) and 2-HJ with m/z 189 (H). (DOCX) [file pone.0091616.s004.docx]

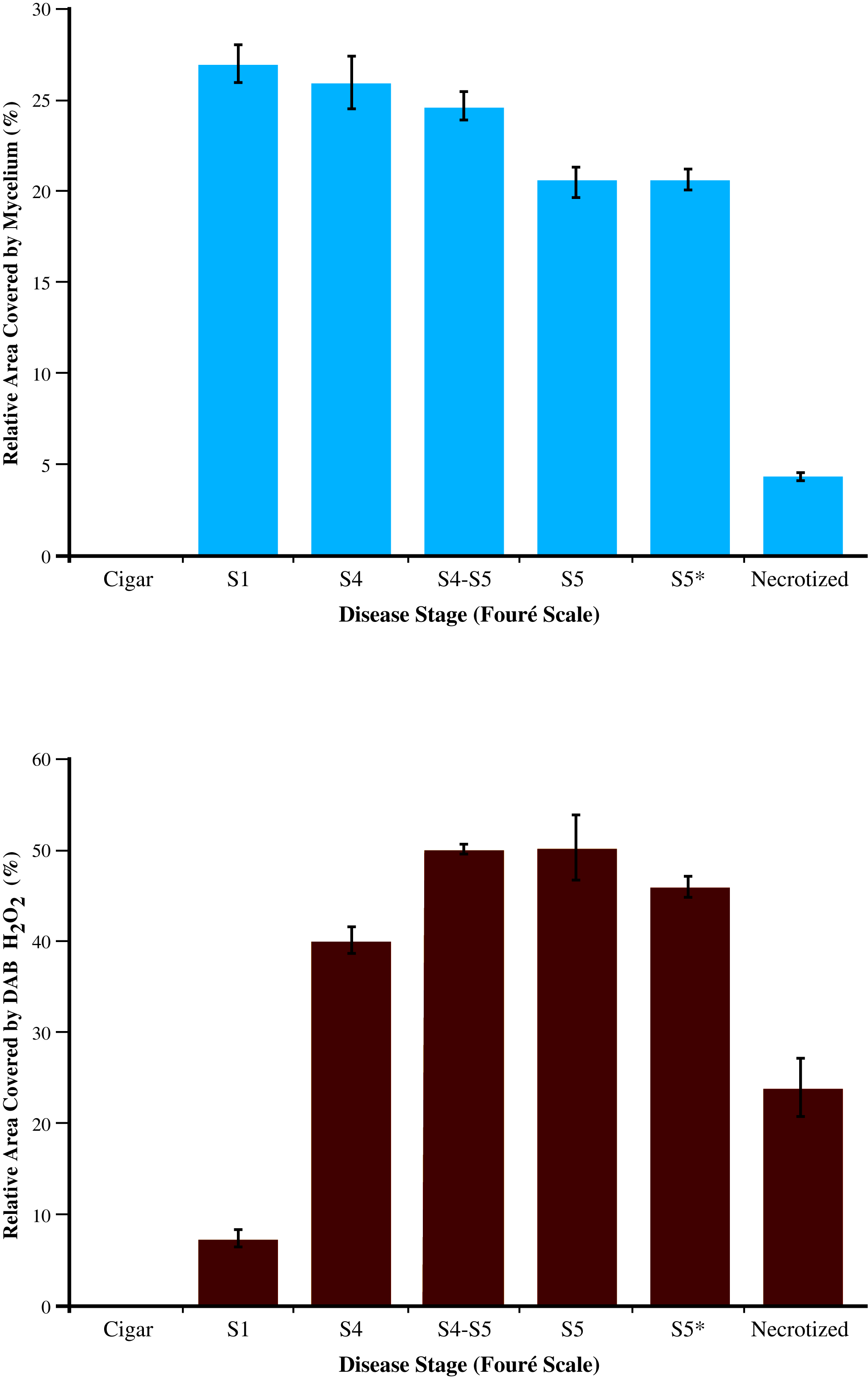


**Figure S5.** Percentage of relative covered area of fungal mycelium and H_2_O_2_ stained with aniline blue and DAB polymerization in presence of endogenous peroxidase at different stage of black Sigatoka symptoms. This figure is complementary to Figure 6 data**.**
