# Supplementary material for: Singlet Molecular Oxygen Generation by Light-Activated DHN-Melanin of the Fungal Pathogen Mycosphaerella fijiensis in Black Sigatoka Disease of Bananas
Source: PLoS One. 2014 Mar 19;9(3):e91616. doi: 10.1371/journal.pone.0091616 (PMC3960117; doi:10.1371/journal.pone.0091616)
Supplement: Figure S5 — Semi-quantitative analysis of the covered area with the fungus (based on incorporation of aniline blue into fungal hyphae) and the presence of H2O2 in the plant tissue (detected as a reddish-brown color) in each of the stages of the disease. The values were normalized to “capote” leaf (used as control) that shown fungal infection. Results were obtained from at least five leaves analyzed for each stage. Data are given as mean ± SD. One-way Analysis of Variance (ANOVA) with significance level set at α = 0.05 was used to determinate the statistical difference between Fouré Stages. (DOCX) [file pone.0091616.s005.docx]

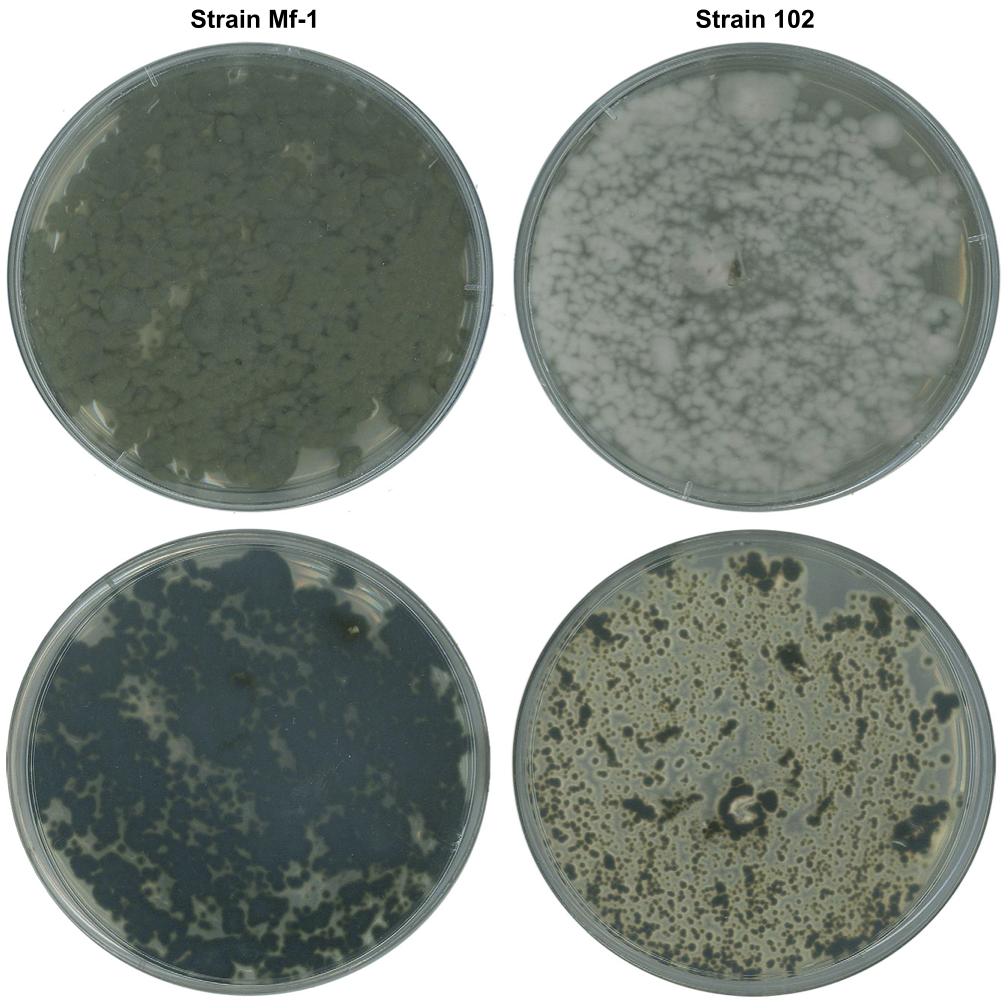


**Figure S3.** Morphological aspects of *M. fijiensis* strains *Mf-1* and *102* used in this study. The fungal strains were cultivated on Potato Dextrose (PDA) and were incubated by 7 days at 27ºC.
