# Supplementary material for: Singlet Molecular Oxygen Generation by Light-Activated DHN-Melanin of the Fungal Pathogen Mycosphaerella fijiensis in Black Sigatoka Disease of Bananas
Source: PLoS One. 2014 Mar 19;9(3):e91616. doi: 10.1371/journal.pone.0091616 (PMC3960117; doi:10.1371/journal.pone.0091616)
Supplement: Table S1 — Elemental analysis of isolated melanins of Mycosphaerella fijiensis. (DOCX) [file pone.0091616.s006.docx]

**Table S1. Elemental analysis of isolated melanins of *Mycosphaerella* fijiensis.**

| **Source** | **% C** | **%H** | **%N** | **%S** |
| --- | --- | --- | --- | --- |
| Mycelium | 46.68 | 4.96 | 2.33 | 0.09 |
| Secreted | 43.88 | 10.27 | 0.34 | 0.08 |
